# The Association between Bisphenol A, Steroid Hormones, and Selected MicroRNAs Levels in Seminal Plasma of Men with Infertility

**DOI:** 10.3390/jcm10245945

**Published:** 2021-12-18

**Authors:** Ewelina Palak, Weronika Lebiedzińska, Sławomir Anisimowicz, Maria Sztachelska, Piotr Pierzyński, Wiesław Wiczkowski, Beata Żelazowska-Rutkowska, Gabriella Nicole Niklińska, Donata Ponikwicka-Tyszko, Sławomir Wołczyński

**Affiliations:** 1 Department of Biology and Pathology of Human Reproduction, Institute of Animal Reproduction and Food Research, Polish Academy of Sciences, 10-748 Olsztyn, Poland; e.palak@pan.olsztyn.pl (E.P.); m.sztachelska@pan.olsztyn.pl (M.S.); d.ponikwicka-tyszko@pan.olsztyn.pl (D.P.-T.); 2 Department of Reproduction and Gynecological Endocrinology, Medical University of Bialystok, 15-089 Białystok, Poland; weronika.lebiedzinska@umb.edu.pl; 3 Gynecology and Reproductive Endocrinology Centre ARTemida, 15-464 Białystok, Poland; sanisimowicz@o2.pl; 4 Oviclinic, 01-377 Warsaw, Poland; piotr.pierzynski@gmail.com; 5 Department of Chemistry and Biodynamics of Food, Institute of Animal Reproduction and Food Research, Polish Academy of Sciences, 10-748 Olsztyn, Poland; w.wiczkowski@pan.olsztyn.pl; 6 Department of Pediatric Laboratory Diagnostic, Medical University of Bialystok, 15-089 Białystok, Poland; beata.zelazowska@umb.edu.pl; 7 Sluzewiec Equine Hospital, 02-684 Warsaw, Poland; g.niklinska@op.pl

**Keywords:** Bisphenol A, seminal plasma, miRNA, steroids

## Abstract

Bisphenol A (BPA), the most common endocrine-disrupting chemical, has been associated with male reproductive dysfunctions. Recently, it has been shown that BPA may also affect miRNAs expression. Herein, we aimed to evaluate the association of BPA levels with steroid hormone concentration and circulating miRNAs levels to investigate the potential direct effect of BPA on homeostasis in the testis environment. The level of BPA in the seminal plasma of azoospermic men was significantly higher compared to the healthy control. The concentrations of estradiol (E2) and androstenedione (A) were significantly decreased in the seminal plasma of azoospermic men compared to the normospermic men. The levels of miR-let-7a, miR-let-7b, and miR-let-7c were significantly up-regulated, and the level of miR-518f was significantly down-regulated in the seminal plasma of the azoospermic men compared to the healthy control. The level of BPA correlated negatively with sperm concentration and normal semen morphology. A significant positive correlation was found between BPA levels and miR-let-7a and miR-let-7c levels, whereas BPA negatively correlated with miR-518f levels. Our results suggest that BPA may negatively affect sperm quality. Moreover, BPA correlated with the miR-let-7a, miR-let-7c, and miR-518f levels in seminal plasma, which suggests that BPA may act directly in seminal plasma, affecting the testicular environment.

## 1. Introduction

Bisphenol A (BPA) is the most common industrial plasticizer classified as an endocrine disrupting-chemical (EDC) [1]. BPA has been found in many plastic products and epoxy resins and has the ability to easily leach into food [2,3]. BPA enters the body through the digestive system, skin contact [4], and inhalation [5], and its presence has been detected in different tissues, serum, urine, seminal plasma, follicular fluid, and umbilical cord plasma [6,7,8]. Exposure to BPA has been shown to have a negative impact on human health, including male reproduction and fertility [7,9,10]. However, studies evaluating the effects of BPA on sperm quality parameters and/or sex hormone levels have been inconsistent [7,11,12,13,14,15,16,17,18].

BPA may alter the function of the hypothalamic–pituitary–testicular axis and cause testicular dysgenesis and atrophy, enlargement of the prostate gland, and changes in semen parameters [9,10,19]. It has been shown that BPA binds with weak affinity to nuclear estrogen receptors (ER) [20] and binds to membrane estrogen receptors [21] with a similar affinity to estradiol (E2). BPA may also exert its effect through the androgen receptors [22], estrogen-related receptors [23], thyroid receptors [24], or glucocorticoid receptors [25]. However, BPA action is not limited to hormonal receptors, but it can also induce several epigenetic modifications in both animals and humans through DNA methylation, histone modifications, and the alteration of microRNA (miRNA) levels [26,27].

MiRNAs are non-coding, single-stranded RNA molecules composed of 18–24 nucleotides [28]. They are important regulators of post-transcriptional gene expression, and they achieve this via translational repression or mRNA degradation in mammals [29]. MiRNAs can be found in a number of different tissues and body fluids, including the testis, sperm, and seminal plasma, and may regulate spermatogenesis, sperm maturation, and capacitation [30,31]. The synthesis and expression of miRNA may be regulated by hormonal signaling [32]. Furthermore, circulating miRNAs may present differential levels under the effects of different toxicants [33]. Differential patterns of miRNAs expression were also induced by EDCs including BPA [27,34]; however, the BPA action in epigenetic modulation is still unclear.

Although the effect of BPA on reproductive tissues is the topic of recent extensive research, there are limited data on how BPA affects the reproductive tissues environment. Seminal plasma is a unique body fluid that creates the environment in which the effects of BPA may be expressed directly in testicular tissue. Herein, we aimed to evaluate the association of BPA levels with steroid hormone concentration and circulating miRNAs levels to investigate the potential direct effect of BPA on homeostasis in the testis environment. The miRNAs chosen for this study were miR-let-7a, miR-let-7b, miR-let-7c, and miR-518f as they have been associated with estrogen and androgen signaling [35,36]. Moreover, we also investigated the possible associations of seminal plasma BPA levels with sperm quality parameters.

## 2. Materials and Methods

### 2.1. Study Participants

The study group comprised of patients recruited in the Department of Reproduction and Gynecological Endocrinology at the Medical University of Bialystok, Poland. The Human Investigation Ethics Committees at the Medical University of Bialystok approved the study. Written informed consent was obtained from all patients prior to inclusion. A total of 116 men with non-obstructive azoospermia (*n* = 20), oligoasthenoteratozoospermia (*n* = 46), and control normospermic (*n* = 50) were enrolled in this study. The mean age and BMI of the men were similar in all groups (Table 1). All of the men included in the study came from cities and had not had high levels of occupational exposure to BPA. Men with pathologies of the epididymis or vas deferens, cryptorchidism, mumps, varicocele, retrograde ejaculation, chromosomal abnormalities, and Y chromosome microdeletions were excluded from the study. Smokers and men who regularly consumed alcohol were also excluded from the study. The controls were healthy men with normal sperm parameters who had fathered at least one healthy child within the past year without assisted reproductive techniques. Patients underwent a standardized ejaculate examination according to the World Health Organization (WHO) criteria. Samples were collected using BPA-free glass equipment. Semen samples were centrifuged for 5 min at 1500× *g* to collect seminal plasma, aliquoted and stored in BPA-free tubes at –80 °C until further analysis.

### 2.2. Semen Analysis

The semen samples were obtained in a private room after a recommended 2–5 days of sexual abstinence. Semen analyses were conducted after liquefaction at 37 °C for 30 min by the micro-cell slide and the computer-aided semen analysis (CASA, version 6.3-SCA^®^, Microptic, S.L., Barcelona, Spain) in accordance with WHO guidelines (World Health Organization. (2010)). WHO laboratory manual for the examination and processing of human semen, 5th ed. World Health Organization. A minimum of 200 sperm cells from at least four different fields were analyzed from each specimen. Each sample was assessed twice. Measured semen parameters included semen volume, sperm concentration, total sperm count, and sperm motility. Sperm morphology was evaluated using the Diff-Quik staining set (Medion Grifols Diagnostics AG, Düdingen, Switzerland). Total sperm count (10^6^) was calculated by multiplying sperm concentration (10^6^/mL) by semen sample volume (mL).

### 2.3. BPA Measurement from the Seminal Plasma Samples

Bisphenol A was measured from seminal plasma samples that were thawed at room temperature. BPA was extracted from the samples three times, using sonication in acetonitrile and vortex mixing. The collected supernatants were first centrifuged for 10 min at 5000× *g* and then evaporated at 37 °C under a nitrogen atmosphere until complete solvent evaporation. The formed solids were then dissolved in methanol and injected into UHPLC microcolumns coupled with a mass spectrometer (TripleTOF 5600 + mass spectrometer, AB SCIEX, Framingham, MA, USA). Deuterated BPA was used as an internal standard. The BPA concentrations were calculated using a calibration curve for concentrations from 0 to 4.3 nmol/L.

### 2.4. Biochemical Analyses

Estradiol, progesterone, and testosterone levels were measured by the immunoanalyzer Cobas e411 (Roche Diagnostic Ltd., Basel, Switzerland) using the Elecsys Estradiol III Gen Kit (#06656021190; Roche Diagnostic Ltd., Basel, Switzerland), the Elecsys Progesterone II Gen Kit (#12145383 190; Roche Diagnostic Ltd., Basel, Switzerland), and the Elecsys testosterone II Gen Kit (#05200067190; Roche Diagnostic Ltd., Basel, Switzerland). Androstenedione concentrations were measured by the immunoanalyzer IMMULITE 1000 (Siemens Healthcare Diagnostics, Chicago, IL, USA) using the IMMULITE 1000 Androstenedione kit (#LKAO1; Siemens Healthcare Diagnostics, Chicago, IL, USA).

### 2.5. Total RNA Isolation

The total RNA was isolated from the seminal plasma using the TRIzol extraction method (Invitrogen, Carlsbad, CA, USA). Three purification steps were added. To achieve technical normalization, all samples were spiked with synthetic non-human *C. elegans* cel-miR-39 (Integrated DNA Technologies, Coralville, IA, USA). The synthetic cel-miR-39 was reconstituted in nuclease-free water (Ambion, Austin, TX, USA), resulting in a 2 × 10^10^ copies/μL stock. Next, the working solution was prepared to provide 1.6 × 10^8^ copies/μL solution. To all samples, 3.5 μL of the cel-miR-39 working solution was added. The RNA concentration was measured using a NanoDrop ND-1000 spectrophotometer (NanoDrop Technologies, Wilmington, DE, USA), while its quality was verified by denaturing agarose gel electrophoresis.

### 2.6. MicroRNA Analysis

cDNA synthesis was performed using the TaqMan^®^MicroRNA Reverse Transcription Kit (Applied Biosystems, Foster City, CA, USA). The reaction was performed with 5 ng of total RNA in a total volume of 15 μL. The mixture was incubated at 16 °C for 30 min, 42 °C for 30 min, and 85 °C for 5 min as recommended by the manufacturer. TaqMan miRNA probes (cel-miR-39, # 000200; U6 snRNA, # 001973; miR-518f-3p, # 002388; miR-let-7a-5p; # 000377; miR-let-7b-5p, # 002619; miR-let-7c-3p; # 002479; Applied Biosystems) were used to assess the selected miRNA expression profile. Real-time PCR assays were performed in a total of 20 μL reaction volume with TaqMan Universal PCR Master Mix, No AmpErase UNG (Applied Biosystems). Each sample was processed in doublets with an initial denaturation at 95 °C for 10 min followed by 40 amplification cycles at 95 °C for 15 s and 60 °C for 60 s using the 7500 Real-Time PCR System (Applied Biosystems). The miRNA Ct-values were normalized using the exogenous cel-miR-39 and endogenous U6 snRNA. After the reactions, the Ct data were determined using default threshold settings and the mean Ct was determined from the duplicate PCRs. The expression of miRNA relative to U6 snRNA was determined using the 2^−ΔCt^ method.

### 2.7. Statistical Analysis

Statistical analyses were conducted using GraphPad Prism 9 (GraphPad Software, San Diego, CA, USA). Due to non-normal distribution of data, the non-parametric Kruskal–Wallis test with Dunn’s post hoc test was applied in the analysis. The correlations between the studied parameters were assessed with Spearman’s correlation coefficient. Statistical significance was assumed at *p* < 0.05.

## 3. Results

**Figure 1:**
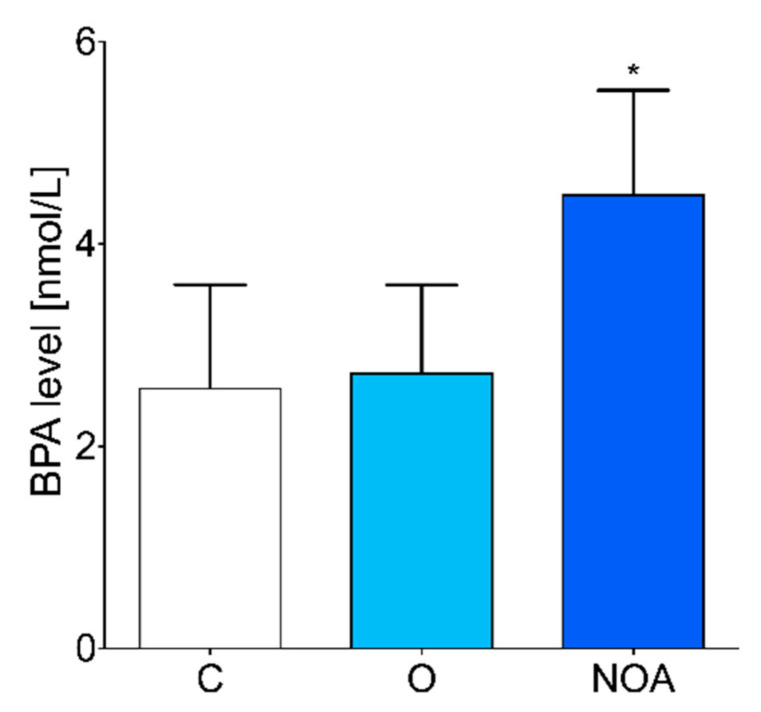
BPA concentrations measured in the seminal plasma of the group of men with non-obstructive azoospermia, the group of men with oligoasthenoteratozoospermia, and the control group of healthy men. The asterisk indicates significant differences between the groups (* *p* < 0.05). C, control; NOA, non-obstructive azoospermia; O, oligoasthenoteratozoospermia.

No significant differences in age and BMI were observed between men with oligoasthenoteratozoospermia, men with azoospermia, and the control (Table 1). The level of BPA in the seminal plasma was significantly higher in the group of men with azoospermia compared to the control group of healthy men (Figure 1). No difference in seminal plasma BPA levels was observed between the group of men with oligoasthenoteratozoospermia compared to the control (Figure 1).

**Figure 2:**
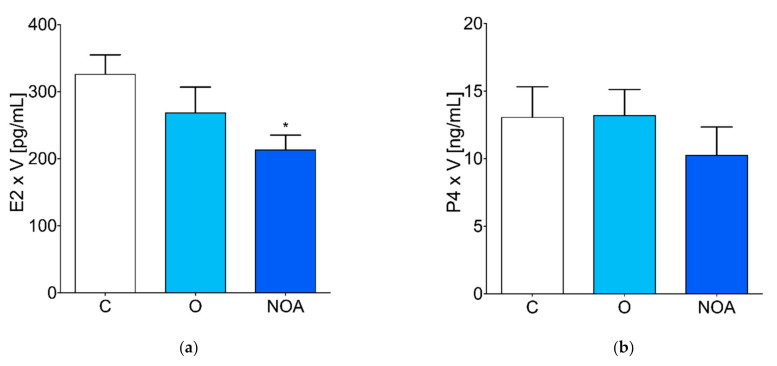

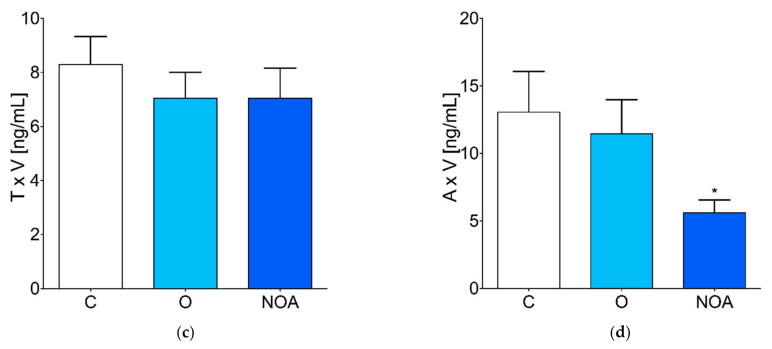
Steroid hormone levels in seminal plasma. (**a**) estradiol, (**b**) progesterone, (**c**) testosterone, and (**d**) androstenedione concentrations measured in the seminal plasma of the group of men with non-obstructive azoospermia, the group of men with oligoasthenoteratozoospermia, and the control group of healthy men. The asterisk indicates significant differences between the groups (* *p* < 0.05). A, androstenedione; C, control; E2, estradiol, NOA, non-obstructive azoospermia; O, oligoasthenoteratozoospermia; P4, progesterone; T, testosterone; V, semen sample volume.

The level of E2 was significantly decreased in the seminal plasma of the group of men with azoospermia compared to the control (Figure 2a). There were no significant differences in the P4 and T concentrations in the seminal plasma of the group of men with azoospermia, oligoasthenoteratozoospermia, and the control (Figure 2b,c). The concentration of A was significantly lower in the seminal plasma of the group of men with azoospermia compared to the control (Figure 2d). The ratio of E2 to T in the control, oligoasthenoteratozoospermia, and azoospermia groups was 62.56 ± 12.35, 51.44 ± 6.38, and 65.45 ± 15.92, respectively.

**Figure 3:**
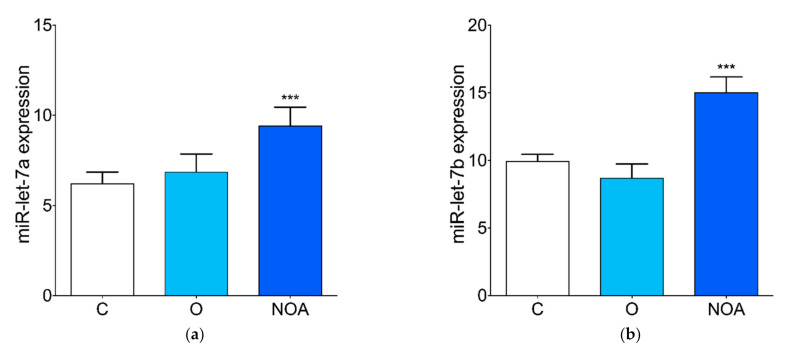

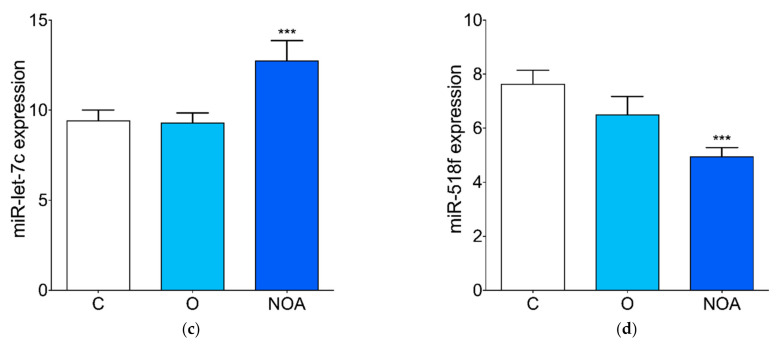
Expression profile of selected miRNAs in seminal plasma. qPCR analysis of the (**a**) miR-let-7a, (**b**) miR-let-7b, (**c**) miR-let-7c, and (**d**) miR-518f expression levels in the seminal plasma of the group of men with non-obstructive azoospermia, the group of men with oligoasthenoteratozoospermia, and the control group of healthy men. Asterisk indicates significant differences between the groups (*** *p* < 0.001). C, control; NOA, non-obstructive azoospermia; O, oligoasthenoteratozoospermia.

We observed higher levels of circulating miR-let-7a, miR-let-7b, and miR-let-7c in the seminal plasma of the group of men with azoospermia compared to the control (Figure 3a–c). No differences in the seminal plasma levels of miR-let-7a, miR-let-7b, and miR-let-7c were found between the group of men with oligoasthenoteratozoospermia compared to the control (Figure 3a–c). The level of miR-518f was significantly down-regulated in the seminal plasma of men with azoospermia compared to the control (Figure 3d). There was no significant difference in the miR-518f levels in the seminal plasma of men with oligoasthenoteratozoospermia compared to the control (Figure 3d).

The level of BPA correlated negatively with sperm concentration, total sperm concentration, and normal semen morphology (*r* = −0.232, *p* = 0.01, *r* = −0.22, *p* = 0.02 and *r* = −0.193, *p* = 0.04; respectively) (Table 2). Significant positive correlation was found between BPA levels and miR-let-7a and miR-let-7c levels (*r* = 0.189, *p* = 0.04 and *r* = 0.249, *p* = 0.007; respectively) (Table 2). The level of BPA correlated negatively with miR-518f levels (*r* = −0.236, *p* = 0.01) (Table 2). No correlations were observed between BPA levels and total and progressive motility, steroid hormone and miR-let-7b levels (Table 2).

## 4. Discussion

BPA exposure has been associated with adverse effects on male reproductive functions, spermatogenesis, and fertility [37,38]. In this study, we showed that higher BPA seminal plasma levels were associated with azoospermia. Previously, high urinary levels of BPA have also been correlated with sexual dysfunction, greater erectile and ejaculatory problems, and decreased sperm concentration and motility [12,39]. Urinary BPA concentration has also had a positive association with sperm DNA damage [40]. However, some studies showed no significant association between BPA urinary levels and altered semen quality parameters [13,41]. We found a negative correlation between BPA seminal plasma levels, sperm concentration, and normal sperm morphology. Our results are consistent with other studies that show that only seminal plasma BPA was negatively associated with sperm concentration, sperm count, and morphology [7,38]. These discrepancies may occur due to different biological fluids being used to measure BPA levels. However, as seminal plasma represents the testicular environment, it seems to be the most applicable biological fluid to assess the impact of BPA on semen parameters. It has been shown that the BPA concentration in blood plasma positively correlated with BPA levels in seminal plasma, but only BPA seminal plasma concentration negatively correlated with semen parameters [7]. Previous studies showed that BPA levels depend on the living environment and the workplace of the studied patients [17,42]. The BPA serum levels were significantly higher in the men living in metropolitan areas than the men living in urban and rural areas [42]. The level of BPA was also significantly higher in infertile men compared to fertile men [42]. Another study showed that the serum levels of BPA in factory workers were significantly increased, which was associated with disordered male sex hormone levels [17]. In our study, all patients came from urban areas and had not had high levels of occupational exposure to BPA.

Since estrogens play an important role in testicular spermatogenesis, several studies have investigated the impact of BPA on estrogen levels and estrogen metabolism [7,13,38]. A significant positive correlation between urinary BPA and E2 plasma levels has been shown in a group of young men [13]. Blood plasma and seminal plasma BPA levels were also positively associated with E2 concentration in infertile men [7,38]. However, some studies showed either no association between urinary BPA and E2 levels [14] or an inverse association [15]. In our studies, we did not find a correlation between BPA seminal plasma levels and estrogens or other steroids; however, the concentration of E2 and A in the seminal plasma of azoospermic men was decreased.

Recently, aberrant miRNAs expression in testicular tissue has been reported [43]. Alterations of the miRNA levels have also been found in biological fluids such as seminal plasma [44,45]. These altered levels of circulating miRNA have been associated with male infertility [44,45]. In our studies, in azoospermic men, we found increased seminal plasma levels of miR-let-7a, miR-let-7b, and miR-let-7c, but decreased seminal plasma levels of miR-518f. The miR-let-7 family represents the most abundant miRNAs present in the testis that may regulate the cell cycle and proliferation [46], but the exact functions are still not fully understood. Our results are in line with other studies that showed a higher level of miR-let-7a in seminal plasma of azoospermic, asthenozoospermic or oligozoospermic men [31,45]. However, in the seminal plasma of asthenozoospermic men, the level of miR-let-7b was decreased but increased in sperm from infertile men [47,48]. Similarly, in infertile men a decreased level of miR-518f has been reported [48]. The level of miR-518f was also increased in the follicular fluid of hyperandrogenic women with polycystic ovary syndrome [36]. In our studies, we found a decreased level of miR-518f in the seminal plasma of azoospermic men who also had decreased levels of androstenedione. We also found a positive correlation between BPA concentration and miR-let-7a and miR-let-7c levels and a negative correlation between BPA concentration and miR-518f levels in seminal plasma. It has been shown that in human breast cancer cells, BPA may influence estrogen receptor activity and down-regulate miR-let-7 expression [27]. Moreover, the miR-let-7 family seems to be under the control of E2, as the expression of eight members of the miR-let-7 family showed to be up-regulated by E2 in breast cancer cells [35]. An in vivo study on a sheep model showed that BPA down-regulated expression of 45 miRNAs in the fetal ovary, including miR-let-7 family members and up-regulated Cyp19 expression [49]. The predicted bioinformatic functional analysis also showed that Cyp19 might be a target gene for the miR-let-7 family [49]. Moreover, it has been shown that in the endometrial cells from women with endometriosis, inhibition of aromatase significantly increased the expression of miR-let-7 [50]. Thus, it is possible that BPA may regulate the Cyp19 expression through miR-let-7 epigenetic mechanisms [49]. However, the potential molecular mechanism of BPA-CYP19-miR-let-7 signaling is still unknown. It seems that in the seminal plasma and testis, BPA may also affect the miR-let-7 family expression and affect estrogen and androgen signaling; however, the data pertaining to the miR-let-7 family function in testis remain very limited.

## 5. Conclusions

In conclusion, our findings showed that BPA may have different concentrations in seminal plasma and may negatively correlate with sperm quality parameters. It seems that BPA may act directly in seminal plasma, thus affecting the testicular environment and the levels of hormones and miRNAs. However, the exact direct mechanism of BPA action and its interaction with miRNAs in seminal plasma requires further investigation.

## Figures and Tables

**Table 1:** Characteristics of the semen parameters of men included in the study.

Characteristics	Control (*n* = 50)	Oligoasthenotera-Tozoospermia (*n* = 46)	Azoospermia (*n* = 20)	*p*-Value
Age (years)	31.30 ± 0.77	31.36 ± 0.86	31.20 ± 1.22	0.966
BMI	23.92 ± 0.35	23.69 ± 0.34	23.3 ± 0.52	0.608
Volume	4.01 ± 0.17	4.32 ± 0.32	3.75 ± 0.26	0.675
Sperm concentration (×10^6^/mL)	42.56 ± 4.93	5.60 ± 0.72	0	**0.0001**
Total sperm concentration	165.85 ± 19.80	21.28 ± 2.85	0	**0.0001**
Total motility (%)	74.02 ± 1.83	49.03 ± 3.02	0	**0.0001**
Progressive motility (%)	51.45 ± 2.04	28.84 ± 2.54	0	**0.0001**
Normal morphology (%)	12.20 ± 1.26	3.72 ± 0.04	0	**0.0001**

Data are mean ± SEM. If significant, *p*-values are highlighted in bold.

**Table 2:** Pearson’s correlation coefficients of seminal plasma BPA levels with semen quality parameters, steroids, and selected miRNAs.

	BPA	
Parameter	*r*	*p*-value
Sperm concentration	**−0.232**	**0.01**
Total sperm concentration	**−0.22**	**0.02**
Total motility	−0.148	0.11
Progressive motility	−0.068	0.47
Normal morphology	**−0.193**	**0.04**
E2	−0.146	0.12
P4	−0.061	0.51
T	−0.001	0.99
A	−0.125	0.18
miR-let-7a	**0.189**	**0.04**
miR-let-7b	0.105	0.26
miR-let-7c	**0.249**	**0.007**
miR-518f	**−0.236**	**0.01**

If significant, coefficients and *p*-values are highlighted in bold.

## Data Availability

The data presented in this study are available on request from the corresponding author.
